# The application and clinical translation of the self-evolving machine learning methods in predicting diabetic retinopathy and visualizing clinical transformation

**DOI:** 10.3389/fendo.2024.1429974

**Published:** 2024-09-19

**Authors:** Binbin Li, Liqun Hu, Siqing Zhang, Shaojun Li, Wei Tang, Guishang Chen

**Affiliations:** ^1^ Department of Ophthalmology, Ganzhou people’s Hospital, Ganzhou, China; ^2^ Department of Endocrinology, Ganzhou people’s Hospital, Ganzhou, China

**Keywords:** diabetic retinopathy, self-evolving machine learning, diagnostic prediction, visualizing clinical transformation, artificial intelligence

## Abstract

**Objective:**

This study aims to analyze the application and clinical translation value of the self-evolving machine learning methods in predicting diabetic retinopathy and visualizing clinical outcomes.

**Methods:**

A retrospective study was conducted on 300 diabetic patients admitted to our hospital between January 2022 and October 2023. The patients were divided into a diabetic retinopathy group (n=150) and a non-diabetic retinopathy group (n=150). The improved Beetle Antennae Search (IBAS) was used for hyperparameter optimization in machine learning, and a self-evolving machine learning model based on XGBoost was developed. Value analysis was performed on the predictive features for diabetic retinopathy selected through multifactor logistic regression analysis, followed by the construction of a visualization system to calculate the risk of diabetic retinopathy occurrence.

**Results:**

Multifactor logistic regression analysis revealed that being male, having a longer disease duration, higher systolic blood pressure, fasting blood glucose, glycosylated hemoglobin, low-density lipoprotein cholesterol, and urine albumin-to-creatinine ratio were risk factors for the development of diabetic retinopathy, while non-pharmacological treatment was a protective factor. The self-evolving machine learning model demonstrated significant performance advantages in early diagnosis and prediction of diabetic retinopathy occurrence.

**Conclusion:**

The application of the self-evolving machine learning models can assist in identifying features associated with diabetic retinopathy in clinical settings, enabling early prediction of disease occurrence and aiding in the formulation of treatment plans to improve patient prognosis.

## 1 Introduction

As lifestyles evolve, the incidence of diabetes in China is escalating rapidly. This condition is typified by compromised insulin secretion or functionality, with elevated blood glucose levels serving as the principal clinical indicator. Chronic hyperglycemia can precipitate microvascular complications, significantly deteriorating patient outcomes (1, 2). Diabetic retinopathy, a prevalent and severe microvascular sequel of diabetes, is most commonly observed in individuals with type 2 diabetes. This complication can impair the retinal microvascular network, leading to capillary engorgement, disruption of the blood-retinal barrier, exudation, macular edema, and visual impairment. If unaddressed, these changes may progress, causing distortion of the retinal microvasculature, retinal detachment, and potentially culminating in blindness (3, 4). Diabetic retinopathy is associated with factors such as vitreous or preretinal hemorrhage, macular edema, and alterations in macular pigment. It primarily stems from chronic hyperglycemia-induced destabilization of the retinal vascular system, characterized by occlusive circulatory disorders in the retinal vessels (5, 6). Research indicates that the duration of diabetes, patient age, glycemic control, treatment modalities, and other factors significantly influence the development of diabetic retinopathy. Consequently, proactive early screening, identification of pertinent risk factors, and educating patients on preventive measures and treatment options are imperative for mitigating disease progression and reducing the risk of vision loss (7, 8).

In recent years, numerous studies, both domestic and international, have employed generalized linear models to develop predictive models for diabetic retinopathy, demonstrating robust predictive capabilities and clinical efficacy. With the swift progression of machine learning, continuously evolving algorithms have significantly advanced artificial intelligence technologies. Self-evolving machine learning has been effectively applied in the screening and diagnosis of various ophthalmic conditions, including retinal diseases, glaucoma, cataracts, and corneal lesions. This approach minimizes human involvement in data preprocessing, feature engineering, model selection, and parameter tuning by automating the modeling processes (9, 10). The distinct advantage of the self-evolving machine learning resides in its ability to adapt and enhance its predictive accuracy over time, reflecting the dynamic progression of the diseases it aims to forecast. This transformative capability holds the potential to revolutionize early screening and prognosis of diabetic retinopathy, signaling a paradigm shift in the management of this challenging condition. The importance of this research extends beyond its technical innovation, focusing critically on its potential to redefine clinical practices by promoting a proactive and personalized approach to patient care. By exploring the application and clinical translation of the self-evolving machine learning methods, this study seeks to pave the way for more effective, personalized interventions, thereby reshaping the management landscape of diabetic retinopathy. Through this investigation, we aim not only to highlight the significance of these innovative methods but also to usher in a new era of predictive modeling that prioritizes clinical impact and patient outcomes.

## 2 Materials and methods

### 2.1 Subjects

**Figure 1:**
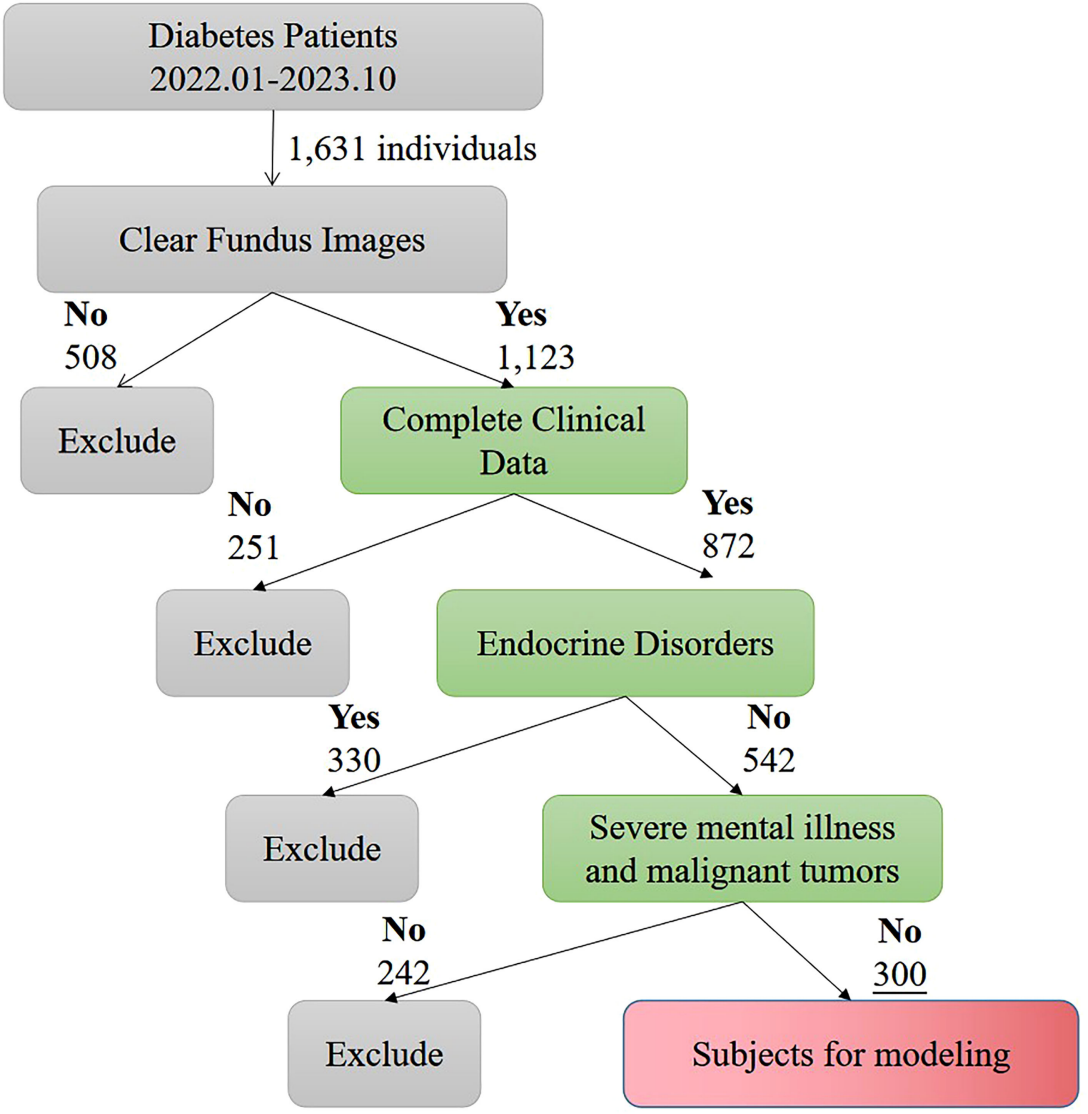
Flow chart of patient enrollment.

A cohort of 300 diabetic patients admitted to our hospital from January 2022 to October 2023 was selected for a retrospective study. The flow of participant selection is detailed in **Figure 1**. Patients were stratified into two groups: the Diabetic Retinopathy Group (n=150) and the Non-Diabetic Retinopathy Group (n=150), based on the presence or absence of diabetic retinopathy. **Figure 1** provides a detailed comparison of baseline characteristics between these groups. Informed consent was obtained from all participants and their families, and the study was conducted with the approval of the hospital’s ethics committee.

Inclusion criteria: 1) Diagnosis consistent with the Diabetes Management Consensus (11) or the Clinical Practice Guidelines for Diabetic Retinopathy (12); 2) Clear fundus examination images; 3) Complete clinical data.

Exclusion criteria: 1) Presence of other endocrine disorders; 2) Presence of severe mental illness; 3) Presence of malignant tumors.

### 2.2 Methods

#### 2.2.1 Clinical data

Clinical data were collected for all participating patients, encompassing 19 indicators: gender, age, duration of diabetes, smoking history, history of alcohol consumption, non-pharmacological treatments, serum creatinine, systolic blood pressure, diastolic blood pressure, fasting blood glucose, 2-hour postprandial blood glucose, body mass index, glycosylated hemoglobin, high-density lipoprotein cholesterol, low-density lipoprotein cholesterol, total cholesterol, triglycerides, blood urea nitrogen, and the urine albumin-to-creatinine ratio. Prior to model construction, all indicator data underwent Z-score normalization to mitigate the impact of dimensional disparities and ensure uniformity in data analysis.

#### 2.2.2 Algorithm improvement

The evolutionary strategy adopted in this study involves a novel swarm intelligence optimization algorithm known as the Beetle Antennae Search (BAS) algorithm. To enhance its global optimization capabilities, three significant improvements have been integrated into the BAS:

Piecewise Chaotic Mapping: The initialization of the swarm intelligence algorithm with chaotic mapping presents multiple advantages:

Enhanced global search capability: The initial population generated through chaotic mapping spans the entire solution space, enhancing the algorithm’s ability to conduct a comprehensive global search and more effectively locate the global optimum.

Accelerated convergence speed: The uniform distribution of the population through chaotic mapping prevents entrapment in local optima, thereby enhancing the algorithm’s convergence rate.

Ensured solution diversity: The high randomness and uncertainty of the population produced by chaotic mapping ensure a diverse set of solutions, increasing the chances of achieving the global optimum (13, 14).

Adaptive t-Distribution: The introduction of a t-distribution mutation operator, with the degrees of freedom parameter linked to the iteration count, perturbs the positions of solutions. This adaptation allows the algorithm to exhibit robust global exploration capabilities in the initial iterations and refined local exploration in later stages, thus boosting the convergence speed.

Random Walk: The random walk strategy, a mathematical statistical model, simulates a path generated by irregular movements. The primary method involves randomly selecting a neighboring point of the current solution for comparison. If this new point is superior, it replaces the current solution as the new focal point. If no better solution is found after N consecutive attempts, the algorithm assumes that the optimal solution is within an N-dimensional sphere centered around the current best solution with the current step size. The process concludes if the step size is below a predetermined threshold; otherwise, the step size is halved, and a new random walk cycle commences. Throughout this iterative process, under specific conditions, the probability distribution converges, resulting in a stable probability distribution (15, 16).

By integrating these enhancements, the Improved Beetle Antennae Search algorithm (IBAS) was developed, combining adaptive t-distribution and random walk strategies to significantly improve performance.

#### 2.2.3 Performance simulation testing of swarm intelligence algorithms

The optimization efficacy of the IBAS algorithm was evaluated using a suite of standard test functions, selecting 23 widely recognized benchmarks. These functions, designed for minimization problems, vary in dimensions and complexity. The primary attributes of these 23 test functions include their search space boundaries, dimensionalities, categories, and known optimal solutions. The functions are categorized into two types: unimodal (U) and multimodal (M). Unimodal functions, which possess a single optimal value, are utilized to gauge the local exploitation capabilities of optimization algorithms. In contrast, multimodal functions, characterized by multiple optima, are used to assess the global exploration abilities of the algorithms.

To ensure a fair comparison of the optimization capabilities before and after the algorithm enhancements, the experimental setup was standardized. The population size was maintained at 30, the number of iterations was fixed at 200, and each configuration was repeated 30 times. The results were analyzed by plotting the average convergence curves from these 30 repetitions, providing a comprehensive view of the algorithm’s performance across different scenarios.

#### 2.2.4 Model construction and evaluation

##### 2.2.4.1 Basic model training

We divided the dataset into a training set (80%, 240 cases) and a test set (20%, 60 cases), where the training dataset underwent five-fold cross-validation. Initially, we employed standard hyperparameter optimization methods (grid search) to run Logistic Regression (LR), Support Vector Machine (SVM), and XGBoost. Grid search is a common hyperparameter optimization method that systematically tests every combination of parameters through a predefined grid. The specific parameters and ranges for hyperparameter optimization are refer to **Table 1** for details.

**Table 1:** Specific parameters and range table of hyperparameter optimization for each model.

Model category	Optimizing hyperparameters	Optimal range	Grid search step size
LR	Regularization parameter	[0.01, 100]	10
	Type of punishment	L1 regularization or L2 regularization	1
SVM	Parameter of penalty	[0.01, 100]	10
	Parameter of Gamma	[0.01, 100]	10
XGBoost	Rate of learning	[0.01, 1]	0.1
	Maximum depth	[1, 20]	0.5
	Maximum number of iterations	[1, 100]	10

LR, Logistic regression; SVM, Support vector machine; XGBoost, eXtreme Gradient Boosting; Grid search tests each predefined combination of parameters using exhaustive methods to find the optimal model configuration, where ‘Grid search step size’ represents the interval between parameter values.

##### 2.2.4.2 The self-evolving machine learning

The self-evolving machine learning is a method that features self-evolution and self-optimization capabilities. Traditional machine learning algorithms typically require manual parameter adjustment and model updates during training. In contrast, the self-evolving machine learning integrates evolutionary computing, genetic algorithms, or other evolutionary methods, enabling the machine learning system to automatically adapt and improve its models and strategies. The best-performing model from the basic models is selected for improvement to construct the self-evolving machine learning model. The optimization parameters are consistent with those listed above, and the specific hyperparameter optimization is as follows: (i) Initialization: IBAS starts with a set of initial candidate solutions, which are randomly generated from the search space of the aforementioned hyperparameters. (ii) Fitness Evaluation: For each candidate solution, the base model is trained using these hyperparameters and evaluated through cross-validation to serve as the fitness value. (iii) Iterative Optimization: IBAS updates the candidate solutions by simulating swarm intelligence behaviors, such as foraging behavior and information sharing, gradually approaching the optimal solution. In each iteration, the algorithm selects and updates candidate solutions based on fitness values, exploring better hyperparameter combinations. (iv) Termination Condition: The algorithm terminates and outputs the optimal hyperparameter combination when a preset number of iterations is reached or when there is no significant improvement in fitness values.

##### 2.2.4.3 Model evaluation and comparison

For performance assessment, we utilized multiple metrics, including Precision (PRE), Sensitivity (SEN), Specificity (SPE), Accuracy (ACC), F1 Score (F1), Area Under the Receiver Operating Characteristic Curve (ROC-AUC), and Area Under the Precision-Recall Curve (PR-AUC). These metrics help us evaluate the predictive power and practicality of the models from various perspectives. To reduce the risk of overfitting and enhance the stability of training, we implemented five-fold cross-validation within the training dataset.

### 2.3 Statistical analysis

Statistical analysis of the data was conducted using SPSS 25.0 software. A significance level of P<0.05 was used to indicate statistical significance. Categorical data were presented as [n (%)] and compared using the chi-square test. Continuous variables with a normal distribution were presented as (mean ± standard deviation) and compared using the t-test.

## 3 Results

### 3.1 Analysis of influencing factors of diabetic retinopathy

#### 3.1.1 Univariate analysis

In the diabetic retinopathy group and non-diabetic retinopathy group, there were statistically significant differences (P<0.05) in gender, age, duration of diabetes, smoking history, non-pharmacological treatment, systolic blood pressure, fasting blood glucose, 2-hour postprandial blood glucose, glycosylated hemoglobin, high-density lipoprotein cholesterol, low-density lipoprotein cholesterol, blood urea nitrogen, and urine albumin/creatinine ratio. Refer to **Table 2** for details.

**Table 2:** Univariate analysis of diabetic retinopathy.

Variables of interest	Diabetic retinopathy group (n=150)	Non-diabetic retinopathy group (n=150)	*t*/*χ* ^2^	*P*
Gender (n)			14.599	<0.001
male	97 (64.67)	64 (42.67)		
female	53 (35.33)	86 (57.33)		
Age (years)	64.18 ± 8.43	60.87 ± 9.04	3.280	0.001
Duration of diabetes (years)	10.87 ± 6.01	9.11 ± 5.19	2.715	0.007
Smoking history (n)			4.975	0.026
Yes	71 (47.33)	52 (34.67)		
No	79 (52.67)	98 (65.33)		
Drinking history (n)			0.298	0.585
Yes	37 (24.67)	33 (22.00)		
No	113 (75.33)	117 (78.00)		
Non-pharmacological treatment (n)			18.274	<0.001
No	96 (64.00)	59 (39.33)		
Yes	54 (36.00)	91 (60.67)		
Serum creatinine (μmol/L)	82.13 ± 33.19	77.65 ± 41.06	1.039	0.300
Systolic Blood Pressure (mmHg)	140.18 ± 19.83	135.23 ± 15.74	2.395	0.017
Diastolic Blood Pressure (mmHg)	83.12 ± 9.13	82.49 ± 9.58	0.583	0.560
Fasting blood glucose (μmol/L)	7.84 ± 2.15	7.33 ± 1.76	2.248	0.025
2-hour postprandial blood glucose (μmol/L)	12.45 ± 2.43	11.88 ± 1.81	2.304	0.022
Body mass index (kg/m2)	25.41 ± 3.41	25.56 ± 3.61	0.370	0.712
Glycosylated hemoglobin (%)	7.43 ± 1.31	6.11 ± 0.75	10.710	<0.001
High density lipoprotein cholesterol (mmol/L)	1.31 ± 0.81	1.91 ± 1.05	5.541	<0.001
Low-density lipoprotein cholesterol (mmol/L)	3.71 ± 1.13	3.43 ± 0.83	2.446	0.015
Total cholesterol (mmol/L)	5.48 ± 1.31	5.52 ± 1.04	0.293	0.770
Triglyceride (mmol/L)	2.84 ± 1.03	2.71 ± 0.99	1.114	0.266
Blood urea nitrogen (mmol/L)	5.45 ± 1.74	5.01 ± 1.66	2.241	0.026
Urinary albumin/creatinine ratio (mg/g)	21.24 ± 6.28	6.32 ± 2.76	26.638	<0.001

#### 3.1.2 Multivariate analysis

A multiple logistic regression analysis was performed with the occurrence of diabetic retinopathy (present=1, absent=0) as the dependent variable and the significant variables from the univariate analysis as independent variables. The analysis used the original values for continuous data and set the reference group for categorical variables. The results indicated that being male, having a longer duration of diabetes, higher systolic blood pressure, fasting blood glucose, glycosylated hemoglobin, low-density lipoprotein cholesterol, and urine albumin/creatinine ratio were identified as risk factors for developing diabetic retinopathy in diabetic patients. Non-pharmacological treatment was identified as a protective factor for the development of diabetic retinopathy. Refer to **Table 3** for further details.

**Table 3:** Multivariate analysis of diabetic retinopathy.

Variables of interest	Reference group	β	SE	Wald	P	*OR*	95%CI
Gender							
male	female	1.132	0.208	29.619	0.000	3.102	2.694~3.510
Yes	No						
Non-pharmacological treatment							
Yes	No	-0.642	0.207	9.619	0.002	0.526	0.121~0.932
Duration of diabetes	—	0.512	0.236	4.707	0.034	1.669	1.206~2.131
Systolic Blood Pressure	—	0.982	0.418	5.519	0.019	2.670	1.851~3.489
Fasting blood glucose	—	0.046	0.019	5.861	0.016	1.047	1.010~1.084
Glycosylated hemoglobin	—	0.992	0.362	7.509	0.006	2.697	1.987~3.406
Urinary albumin/creatinine ratio	—	0.072	0.027	7.111	0.008	1.075	1.022~1.128
Low-density lipoprotein cholesterol	—	0.672	0.176	14.579	0.000	1.958	1.613~2.303
Constant quantity	—	-0.972	0.178	29.819	0.000		

### 3.2 Precise predictive results of the self-evolving machine learning model

#### 3.2.1 Performance testing of the improved optimization algorithm

**Figure 2:**
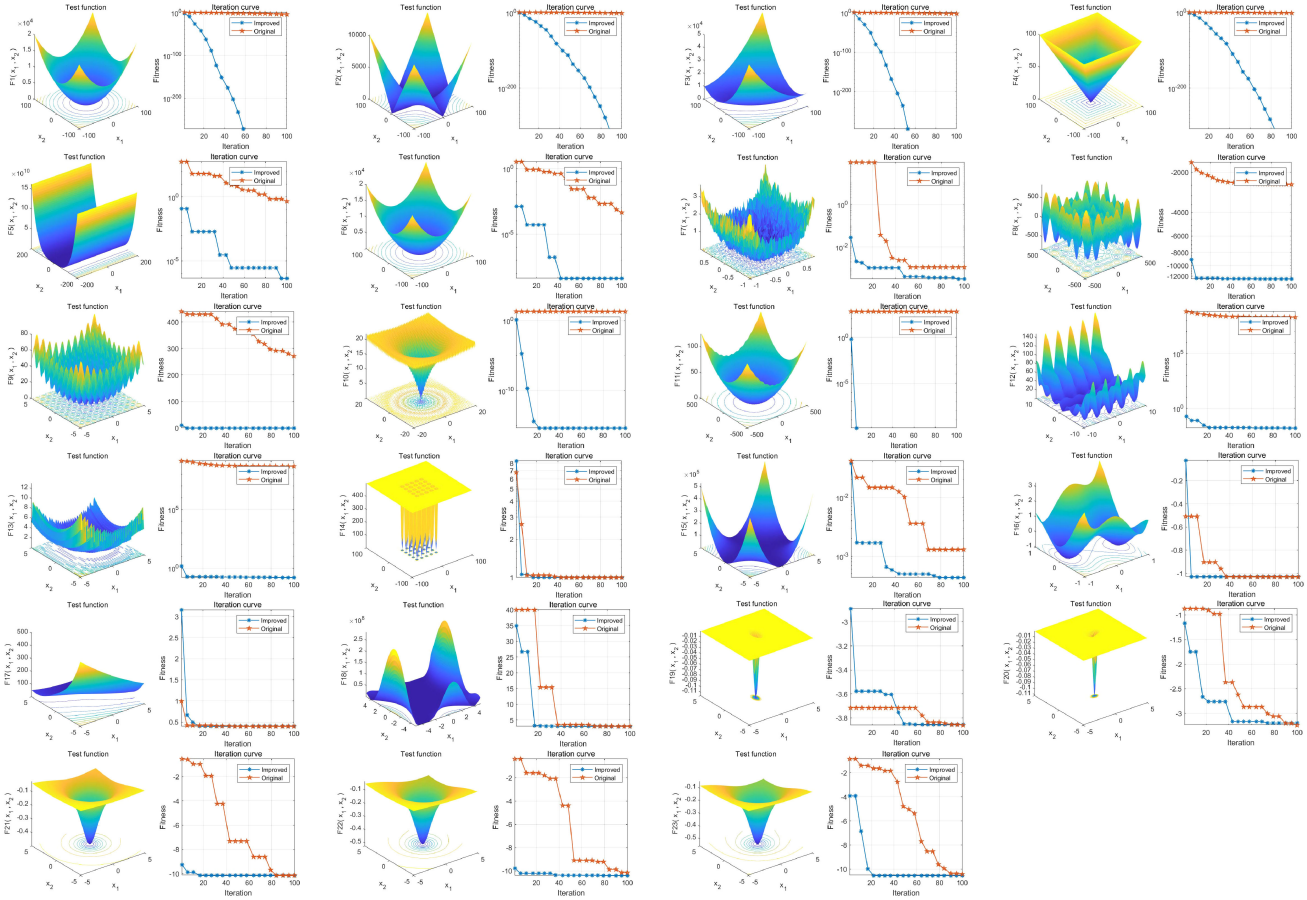
Performance analysis of IBAS on 23 benchmark functions. The three-dimensional surface plots in the figure depict the two-dimensional search space for each benchmark function. The convergence curves demonstrate the convergence trends of the first solution dimension for each benchmark function, comparing the trends between BAS (blue) and the improved IBAS (red).

23 common test functions were used to test the BAS before and after the improvement. The results showed a significant improvement in the overall convergence and global optimization capability of IBAS compared to the previous version, as shown in **Figure 2**.

#### 3.2.2 Construction of the self-evolving machine learning model

**Figure 3:**
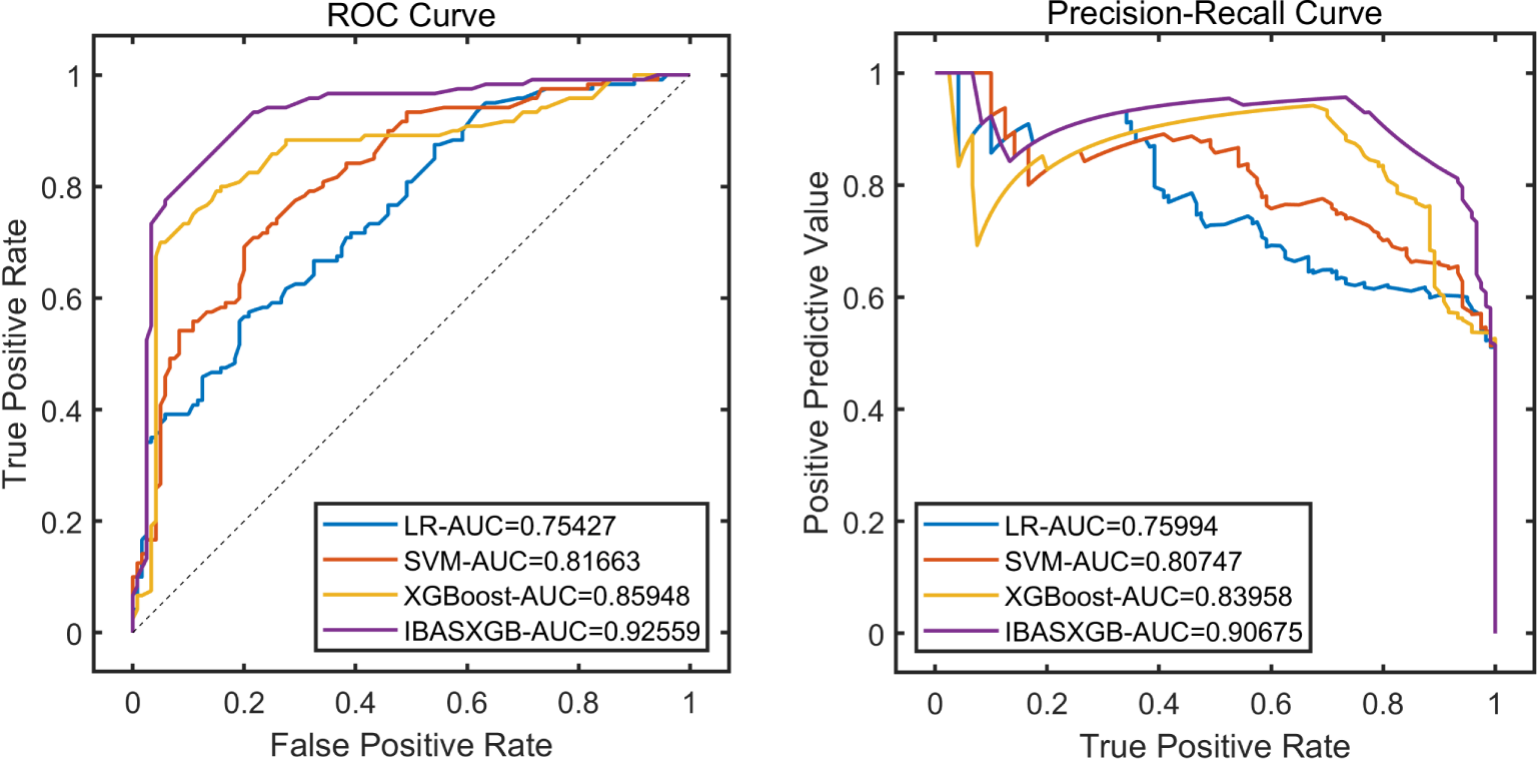
Performance analysis of the training set.

The three base models were tested according to standard hyperparameter optimization methods, and the XGBoost with the best results was finally selected as the base model for our study. Based on the results of the multiple logistic regression analysis, the selected variables were used as independent variables, and the occurrence of diabetic retinopathy in diabetic patients was used as the dependent variable to construct the machine learning dataset. 80% of the dataset was selected as the training set for model construction. Refer to **Table 4** and **Figure 3** for details.

**Table 4:** Model construction of the training set.

Model	PRE	SEN	SPE	ACC	F1	ROC-AUC	PR-AUC
LR	0.6757	0.6250	0.7000	0.6625	0.6494	0.7543	0.7599
SVM	0.7395	0.7333	0.7417	0.7375	0.7364	0.8166	0.8075
XGBoost	0.8969	0.7250	0.9167	0.8208	0.8018	0.8595	0.8396
IBAS-XGB	0.8644	0.8500	0.8667	0.8583	0.8571	0.9256	0.9068

LR, Logistic regression; SVM, Support vector machine; XGBoost, eXtreme Gradient Boosting; IBAS, Improved Bees Algorithm Search.

#### 3.2.3 Validation of the self-evolving machine learning model

**Figure 4:**
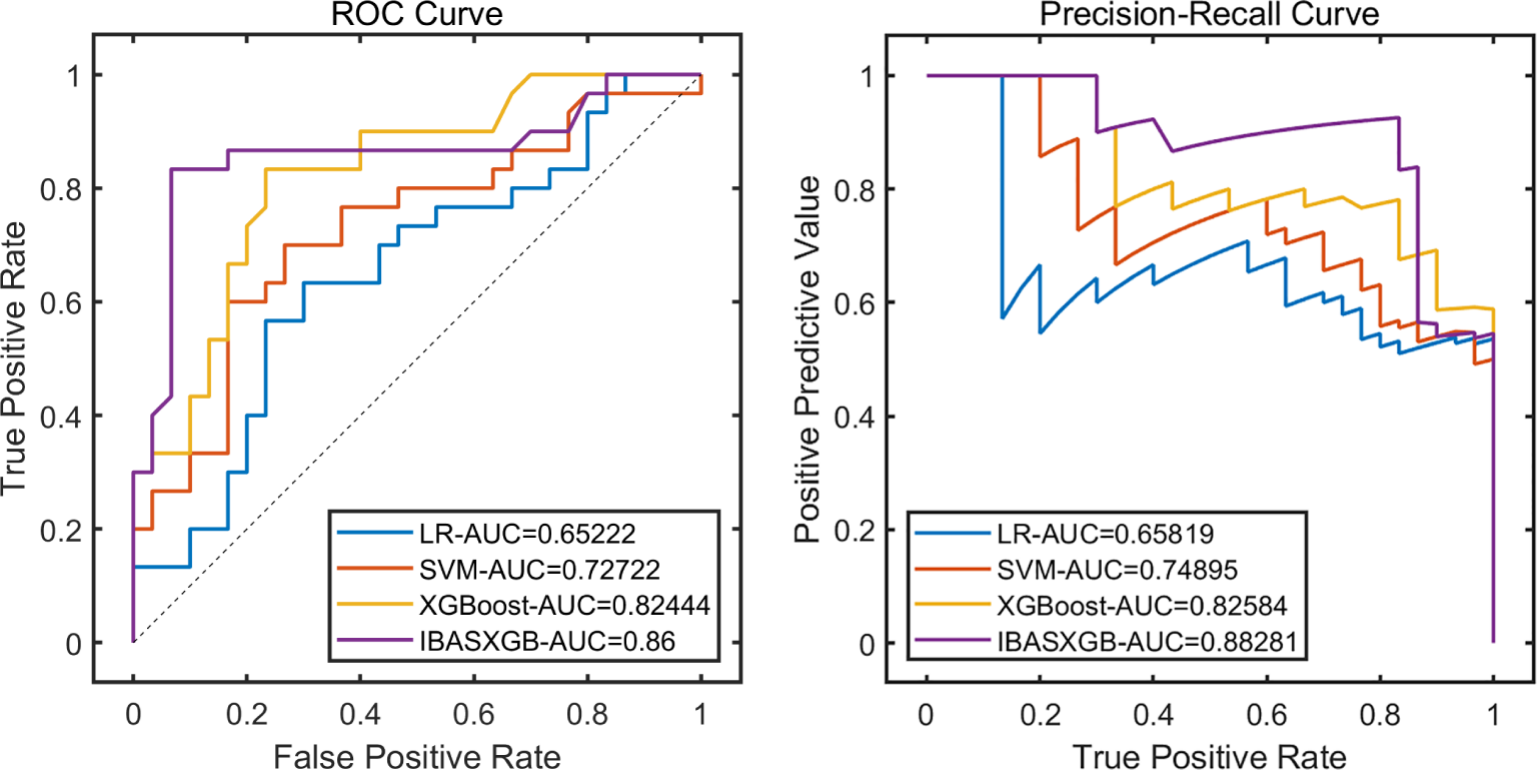
Performance analysis of the test set.

The remaining 20% of the dataset was used as the test set to evaluate the performance of the model. The results demonstrated the significant advantages of the IBAS-XGB self-evolving machine learning model. Please refer to **Table 5** and **Figure 4** for further details.

**Table 5:** Test set model performance verification.

Model	PRE	SEN	SPE	ACC	F1	ROC-AUC	PR-AUC
LR	0.6500	0.4333	0.7667	0.6000	0.5200	0.6522	0.6582
SVM	07308	0.6333	0.7667	0.7000	0.6786	0.7272	0.7490
XGBoost	0.8000	0.6777	0.8333	0.7500	0.7273	0.8244	0.8258
IBAS-XGB	0.8621	0.8333	0.8667	0.8500	0.8475	0.8600	0.8828

LR, Logistic regression; SVM, Support vector machine; XGBoost, eXtreme Gradient Boosting; IBAS, Improved Bees Algorithm Search.

### 3.3 Construction of the visualization software system

The visualization system offers a comprehensive suite of functionalities essential for data preprocessing, high-dimensional data visualization analysis, machine learning model construction, and One-Step analysis:

#### 3.3.1 Data processing

Import Data: Users can upload their dataset by clicking the “Import Data” button. The dataset will then be displayed in the data workspace upon successful import.

Data Cleaning: By clicking the “Data Cleaning” button, users can perform operations such as missing value imputation, outlier removal, normalization, and data balancing to prepare the data for analysis.

High-Dimensional Visualization: This feature allows users to choose their preferred visualization style and generate corresponding graphs by clicking on the “High-Dimensional Visualization” button.

#### 3.3.2 Modeling analysis

Users can select between classification or regression tasks to construct appropriate machine learning models. For integrated feature selection and hyperparameter optimization, users can access the one-step analysis interface via the “One-Step” button. Before initiating the analysis, users must set parameters such as population size, iteration count, test set proportion, and cross-validation folds. Incorrect parameter settings will trigger a warning message. After setting the initial parameters, users can choose their preferred combination of the IBAS algorithm and the machine learning model, then click the “Run” button. The system will verify the successful initialization of parameters and request confirmation to proceed with the analysis. Upon confirmation, the system initiates model construction while simultaneously conducting hyperparameter optimization and feature selection. The performance of the model is evaluated using the test set, and upon completion, the system interface displays the convergence curves, training progress on the training set, and predictive performance on the test set.

#### 3.3.3 Additional features

The “Model Evolution Tips” text box provides insights into the selected features and evaluation metrics for the test set.

**Figure 5:**
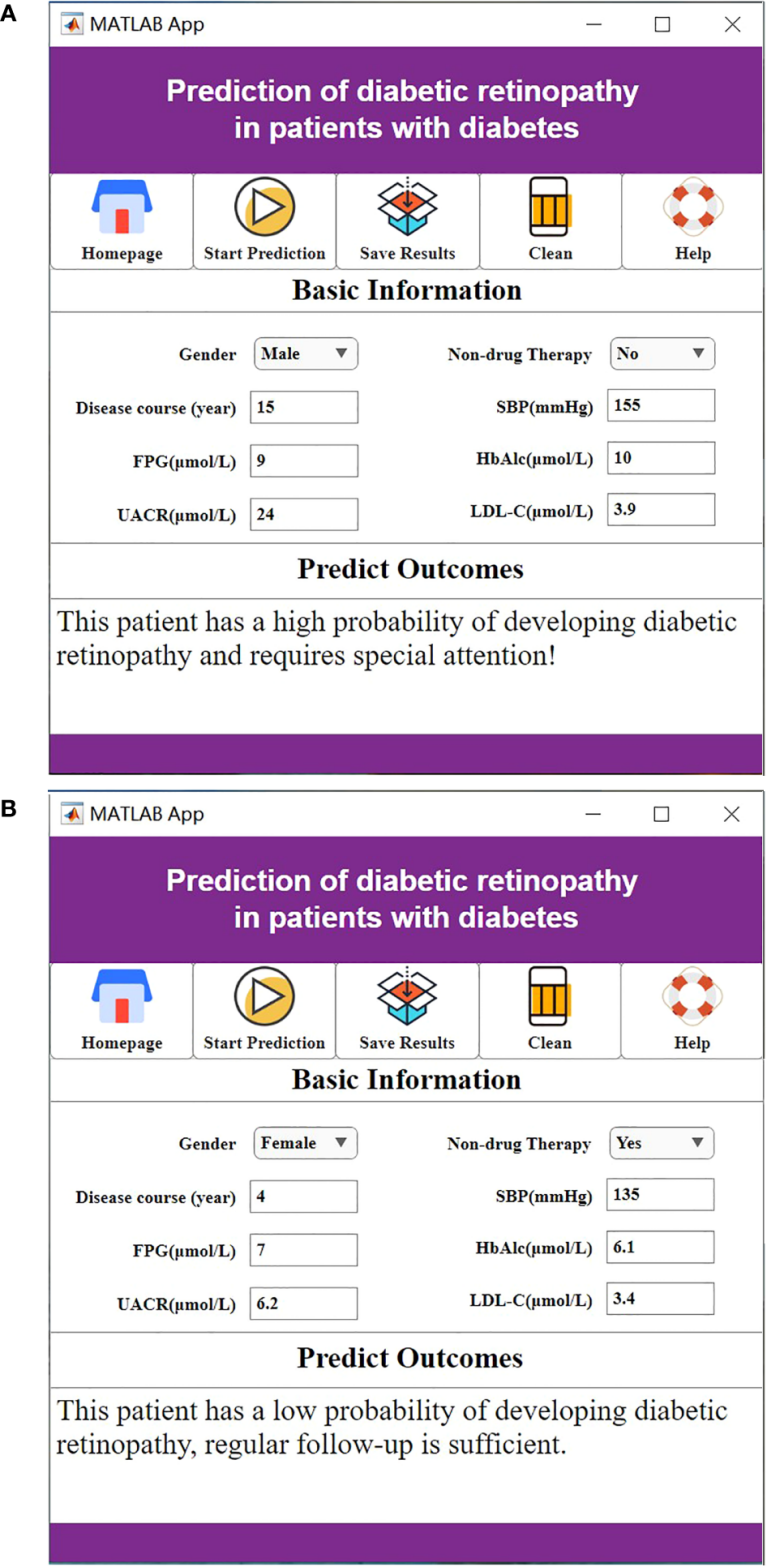
**(A)** The high probability of diabetic retinopathy. **(B)** The low probability of diabetic retinopathy.

For a visual representation of the software system interface, please refer to **Figure 5**.

## 4 Discussion

Prolonged elevation of blood glucose levels can cause endothelial dysfunction, alterations in retinal microvascular circulation, and damage to the retinal barrier. In diabetic patients, metabolic dysfunctions, particularly insulin resistance and dyslipidemia, are known to contribute to the onset of diabetic retinopathy (17, 18). Early screening for diabetic retinopathy presents numerous challenges, with a significant focus on non-invasive methods that utilize clinical and laboratory data for prevention and treatment.

Machine learning, an interdisciplinary domain that merges statistics, various scientific disciplines, and computer technology, facilitates the processing of large datasets. By employing machine learning algorithms, it is possible to identify and select pertinent feature variables from extensive datasets, enhancing the efficiency of the learning process (19, 20). Presently, machine learning finds extensive applications in healthcare, particularly in diagnosing and managing conditions such as glaucoma, cataracts, diabetic retinopathy, retinopathy of prematurity, and retinal vein occlusion (21). In this research, we developed an advanced machine learning model using XGBoost, an evolutionary algorithm. This model streamlines the initial stages of machine learning, including data preparation, encoding, feature selection, extraction, and engineering, thus significantly reducing the complexity involved. The model is capable of autonomously performing algorithm selection, optimization, iteration, and validation with minimal coding required (22, 23). Furthermore, we incorporated the Improved Beetle Antennae Search (IBAS) algorithm for hyperparameter optimization, enhancing the accuracy by leveraging continual improvements from decision tree algorithms. IBAS is an enhanced swarm intelligence optimization algorithm that we have developed, building upon traditional swarm intelligence algorithms. By incorporating new optimization strategies and methods for tuning parameters, we have improved the search efficiency and convergence speed of the algorithm. The core advantage of IBAS lies in its effective exploration and exploitation of the search space, enabling it to identify superior solutions. We employ the IBAS algorithm to optimize the hyperparameters of the XGBoost model. This integration, which we refer to as IBAS-XGB, utilizes the global search capability of IBAS to efficiently explore the hyperparameter space of the XGBoost model. By identifying the most effective parameter combinations, this approach enhances the performance of the model (24). IBAS outperforms standard hyperparameter optimization methods such as grid search mainly because it can more dynamically adapt to and explore the hyperparameter space. Theoretically, if the search range of standard methods is sufficiently broad, they could also find the same hyperparameter combinations discovered by IBAS. However, in practical applications, standard methods may fail to discover these combinations for reasons including: (1) Balance between exploration and exploitation: IBAS can dynamically balance the relationship between exploring new areas and exploiting known good regions. This adaptability often leads to better solutions more quickly than the systematic exploration of space by grid search. (2) Avoiding local optima: In complex hyperparameter spaces, standard methods, especially grid search, are prone to getting stuck in local optima. IBAS, by using multiple search agents that exchange information, can avoid this problem.

This study developed a self-evolving machine learning model based on clinical data and laboratory tests to predict the early risk of diabetic retinopathy. To mitigate randomness and prevent overfitting, five-fold cross-validation was employed. Additionally, model pruning was utilized to enhance performance and accuracy on the test set. Ultimately, eight key factors were identified as significant predictors: male gender, non-pharmacological treatments, prolonged disease duration, elevated systolic blood pressure, fasting blood glucose, glycated hemoglobin, low-density lipoprotein cholesterol, and the urinary albumin-to-creatinine ratio. These predictive factors are influenced by a combination of lifestyle choices, dietary habits, physical inactivity, genetic predispositions, and inadequate insulin secretion in male patients. As diabetes progresses, it exacerbates metabolic dysfunctions such as severe blood glucose fluctuations and dyslipidemia, which have been independently linked to increased risk of diabetic retinopathy (25, 26). Hypertension contributes to damage in retinal microcirculation and the nerve fiber layer, with prolonged high blood pressure leading to endothelial cell damage in capillaries, increased vascular permeability, and subsequent retinal edema and neovascularization. Hyperglycemia exacerbates these effects by thickening the capillary basement membrane, activating the polyol pathway, impairing retinal microcirculation, and accelerating diabetic retinopathy progression (27, 28).

Elevated levels of glycated hemoglobin are implicated in endothelial damage, promoting leukocyte adhesion to endothelial cells and thrombus formation. The oxidative stress and inflammatory states induced by high glucose levels further contribute to endothelial and tissue damage, driving the progression of diabetic retinopathy (29, 30). Hyperlipidemia also plays a role, leading to retinal artery sclerosis and impacting ocular blood supply, thereby accelerating the disease’s progression (31).

The kidneys and retina share similar developmental origins, capillary network structures, and filtration functions. Both are influenced by common factors such as genetics, hemodynamics, and lipid metabolism, with their pathologies involving mechanisms like advanced glycation end-product accumulation, polyol pathway activation, oxidative stress, and inflammatory mediators (32, 33).

Non-pharmacological interventions for diabetes, such as dietary, exercise, and psychological therapies, are crucial. Negative emotional states can exacerbate insulin resistance, possibly through abnormal gene expression and challenges in controlling glucose levels, thus contributing to the development of diabetic retinopathy. The efficacy of psychological interventions and cognitive-behavioral therapy in managing diabetes has been well-documented (34, 35).

Compared to traditional statistical models such as logistic regression, the self-evolving machine learning models offer enhanced accuracy and lower the barriers to adopting artificial intelligence technology through automation. These models enable healthcare professionals to dynamically assess the risk of diabetic retinopathy in diabetic patients and tailor interventions accordingly. For instance, by managing dietary intake to control abnormal blood glucose and blood pressure levels, healthcare providers can offer personalized guidance to patients. This guidance can include recommendations on diet, exercise, blood glucose monitoring, and education on disease prevention (36).

## 5 Conclusion

In summary, the application of the self-evolving machine learning models can help in clinically identifying features of diabetic patients with concurrent retinopathy. This enables early prediction of disease occurrence and assists in formulating treatment plans to improve prognosis. In the future, the self-evolving machine learning models are expected to become essential tools in clinical practice, these models may also play a significant role in the early diagnosis and prediction of other chronic diseases, providing more objective evidence for medical decision-making. With ongoing technological advancements and the accumulation of more clinical data, these self-evolving machine learning methods are poised to bring further innovation and progress to the healthcare field.

However, this study also has certain limitations. One notable limitation of our study is the retrospective nature of the data collection process. As a result, we were reliant on the available historical patient data, which may have inherent biases and missing information. Additionally, the study was conducted at a single center, which could limit the generalizability of our findings to broader populations and healthcare settings. Future studies involving multi-center collaborations and diverse patient populations could offer a more comprehensive understanding of the predictive features and outcomes related to diabetic retinopathy.

Furthermore, while our the self-evolving machine learning model demonstrated promising performance in predicting diabetic retinopathy, it is essential to acknowledge that the model’s predictive accuracy may be influenced by the quality and completeness of the input data. Variability in data collection methods and potential confounding variables not included in our analysis could impact the model’s predictive capabilities. When the Improved Swarm Intelligence Optimization Algorithm (IBAS) is used as a hyperparameter optimization method in XGBoost models, although this approach can enhance model performance, it also has the limitation of high computational costs. IBAS, as a swarm intelligence optimization algorithm, has a relatively high algorithmic complexity, especially on large-scale datasets. During hyperparameter optimization, IBAS requires multiple runs of the XGBoost model to evaluate different parameter combinations. Each run involves the entire training process, which can lead to significant computational costs. This is particularly true in cases where the parameter space is large or the dataset is extensive, making the optimization process very time-consuming. This may not be suitable for applications that require rapid iteration and experimentation. This limitation suggests that when using IBAS to optimize hyperparameters of the XGBoost model, there is a need to balance computational costs and model generalization capabilities to ensure the model’s practicality and effectiveness.

Moreover, the clinical translation of our findings and the implementation of predictive models in real-world healthcare settings may face challenges related to data privacy, interpretability of machine learning algorithms, and integration into existing clinical workflows. These practical considerations represent important limitations that should be carefully addressed in future research and during the implementation of predictive models in clinical practice.

In conclusion, while our study has provided valuable insights into the application of the self-evolving machine learning methods for predicting diabetic retinopathy, we recognize the need for future research to address the limitations highlighted, ensuring the robustness and applicability of predictive models in diverse clinical settings.

## Data Availability

The raw data supporting the conclusions of this article will be made available by the authors, without undue reservation.

## References

[1] Srikanth V Sinclair AJ Hill-Briggs F Moran C Biessels GJ. Type 2 diabetes and cognitive dysfunction-towards effective management of both comorbidities. Lancet Diabetes Endocrinol. (2020) 8:535–45. doi: 10.1016/S2213-8587(20)30118-2 32445740

[2] Artasensi A Pedretti A Vistoli G Fumagalli L. Type 2 diabetes mellitus: A review of multi-target drugs. Molecules. (2020) 25:1987. doi: 10.3390/molecules25081987 32340373 PMC7221535

[3] Fung TH Patel B Wilmot EG Amoaku WM. Diabetic retinopathy for the non-ophthalmologist. Clin Med (Lond). (2022) 22:112–6. doi: 10.7861/clinmed.2021-0792 PMC8966825 35304370

[4] Chandrasekaran PR Madanagopalan VG Narayanan R. Diabetic retinopathy in pregnancy - A review. Indian J Ophthalmol. (2021) 69:3015–25. doi: 10.4103/ijo.IJO_1377_21 PMC8725079 34708737

[5] Li Y Mitchell W Elze T Zebardast N. Association between diabetes, diabetic retinopathy, and glaucoma. Curr Diabetes Rep. (2021) 21:38. doi: 10.1007/s11892-021-01404-5 34495413

[6] Grzybowski A Brona P Lim G Ruamviboonsuk P Tan GSW Abramoff M. Artificial intelligence for diabetic retinopathy screening: a review. Eye (Lond). (2020) 34:451–60. doi: 10.1038/s41433-019-0566-0 PMC7055592 31488886

[7] Ghamdi AHA. Clinical predictors of diabetic retinopathy progression; A systematic review. Curr Diabetes Rev. (2020) 16:242–7. doi: 10.2174/1573399815666190215120435 30767747

[8] Yin L Zhang D Ren Q Su X Sun Z. Prevalence and risk factors of diabetic retinopathy in diabetic patients: A community based cross-sectional study. Med (Baltimore). (2020) 99:19236. doi: 10.1097/MD.0000000000019236 PMC7478682 32118727

[9] Hung AJ Chen J Gill IS. Automated performance metrics and machine learning algorithms to measure surgeon performance and anticipate clinical outcomes in robotic surgery. JAMA Surg. (2018) 153:770–1. doi: 10.1001/jamasurg.2018.1512 PMC9084629 29926095

[10] Leite D Martins A Jr Rativa D De Oliveira JFL Maciel AMA. An automated machine learning approach for real-time fault detection and diagnosis. Sensors (Basel). (2022) 22:6138. doi: 10.3390/s22166138 36015899 PMC9413480

[11] De Boer IH Khunti K Sadusky T Tuttle KR Neumiller JJ Rhee CM. Diabetes management in chronic kidney disease: A consensus report by the american diabetes association (ADA) and kidney disease: improving global outcomes (KDIGO). Diabetes Care. (2022) 45:3075–90. doi: 10.2337/dci22-0027 PMC9870667 36189689

[12] Rajavi Z Safi S Javadi MA Azarmina M Moradian S Entezari M. Diabetic retinopathy clinical practice guidelines: customized for Iranian population. J Ophthalmic Vis Res. (2016) 11:394–414. doi: 10.4103/2008-322X.194131 27994809 PMC5139552

[13] Zheng Y Li L Qian L Cheng B Hou W Zhuang Y. Sine-SSA-BP ship trajectory prediction based on chaotic mapping improved sparrow search algorithm. Sensors (Basel). (2023) 23:704. doi: 10.3390/s23020704 36679503 PMC9864043

[14] Jia J Liu L Liang Y Han Z Wang X. Chaotic mapping-based anti-sorting radio frequency stealth signals and compressed sensing-based echo signal processing technology. Entropy (Basel). (2022) 24:1559. doi: 10.3390/e24111559 36359648 PMC9689734

[15] Caramês LGP Matos YB Bartumeus F Bezerra CG Macrì T da Luz MGE. Lévy walkers inside spherical shells with absorbing boundaries: Towards settling the optimal Lévy walk strategy for random searches. Phys Rev E. (2022) 106:54147. doi: 10.1103/PhysRevE.106.054147 36559395

[16] Valdeolivas A Tichit L Navarro C Perrin S Odelin G Levy N. Random walk with restart on multiplex and heterogeneous biological networks. Bioinformatics. (2019) 35:497–505. doi: 10.1093/bioinformatics/bty637 30020411

[17] Sanz-Cánovas J López-Sampalo A Cobos-Palacios L Ricci M Hernández-Negrín H Mancebo-Sevilla JJ. Management of type 2 diabetes mellitus in elderly patients with frailty and/or sarcopenia. Int J Environ Res Public Health. (2022) 19:8677. doi: 10.3390/ijerph19148677 35886528 PMC9318510

[18] Dugani SB Mielke MM Vella A. Burden and management of type 2 diabetes in rural United States. Diabetes Metab Res Rev. (2021) 37:3410. doi: 10.1002/dmrr.3410 PMC7990742 33021052

[19] Silva GFS Fagundes TP Teixeira BC Chiavegatto Filho ADP. Machine learning for hypertension prediction: a systematic review. Curr Hypertens Rep. (2022) 24:523–33. doi: 10.1007/s11906-022-01212-6 35731335

[20] Jiang T Gradus JL Rosellini AJ. Supervised machine learning: A brief primer. Behav Ther. (2020) 51:675–87. doi: 10.1016/j.beth.2020.05.002 PMC7431677 32800297

[21] Sarhan MH Nasseri MA Zapp D Maier M Lohmann CP Navab N. Machine learning techniques for ophthalmic data processing: A review. IEEE J BioMed Health Inform. (2020) 24:3338–50. doi: 10.1109/JBHI.6221020 32750971

[22] Volpato V Mor-Avi V Narang A Prater D Gonçalves A Tamborini G. Automated, machine learning-based, 3D echocardiographic quantification of left ventricular mass. Echocardiography. (2019) 36:312–9. doi: 10.1111/echo.14234 30592791

[23] El Naqa I Irrer J Ritter TA DeMarco J Al-Hallaq H Booth J. Machine learning for automated quality assurance in radiotherapy: A proof of principle using EPID data description. Med Phys. (2019) 46:1914–21. doi: 10.1002/mp.13433 30734324

[24] Qian Q Deng Y Sun H Pan J Yin J Feng Y. Enhanced beetle antennae search algorithm for complex and unbiased optimization. Soft Comput. (2022) 26:10331–69. doi: 10.1007/s00500-022-07388-y PMC9392993 36034767

[25] Milluzzo A Maugeri A Barchitta M Sciacca L Agodi A. Epigenetic mechanisms in type 2 diabetes retinopathy: A systematic review. Int J Mol Sci. (2021) 22:10502. doi: 10.3390/ijms221910502 34638838 PMC8509039

[26] Lou J Jing L Yang H Qin F Long W Shi R. Risk factors for diabetic nephropathy complications in community patients with type 2 diabetes mellitus in Shanghai: Logistic regression and classification tree model analysis. Int J Health Plann Manage. (2019) 34:1013–24. doi: 10.1002/hpm.2871 31368138

[27] Do DV Han G Abariga SA Sleilati G Vedula SS Hawkins BS. Blood pressure control for diabetic retinopathy. Cochrane Database Syst Rev. (2023) 3:6127. doi: 10.1002/14651858 PMC10049880 36975019

[28] Simó-Servat O Hernández C Simó R. Diabetic retinopathy in the context of patients with diabetes. Ophthalmic Res. (2019) 62:211–7. doi: 10.1159/000499541 31129667

[29] Shah M Farooq A Tariq Y. Relationship between glycosylated hemoglobin levels and contrast sensitivity in people with type 2 diabetes mellitus without diabetic retinopathy. Turk J Ophthalmol. (2022) 52:394–9. doi: 10.4274/tjo PMC9811233 36578209

[30] Zhang B Zhang B Zhou Z Guo Y Wang D. The value of glycosylated hemoglobin in the diagnosis of diabetic retinopathy: a systematic review and Meta-analysis. BMC Endocr Disord. (2021) 21:82. doi: 10.1186/s12902-021-00737-2 33902557 PMC8073908

[31] Bryl A Mrugacz M Falkowski M Zorena K. The effect of hyperlipidemia on the course of diabetic retinopathy-literature review. J Clin Med. (2022) 11:2761. doi: 10.3390/jcm11102761 35628887 PMC9146710

[32] Shityakov S Nagai M Ergün S Braunger BM Förster CY. The protective effects of neurotrophins and microRNA in diabetic retinopathy, nephropathy and heart failure via regulating endothelial function. Biomolecules. (2022) 12:1113. doi: 10.3390/biom12081113 36009007 PMC9405668

[33] Sattar N Lee MMY Kristensen SL Branch KRH Del Prato S Khurmi NS. Cardiovascular, mortality, and kidney outcomes with GLP-1 receptor agonists in patients with type 2 diabetes: a systematic review and meta-analysis of randomised trials. Lancet Diabetes Endocrinol. (2021) 9:653–62. doi: 10.1016/S2213-8587(21)00203-5 34425083

[34] Nguyen C Sirineni G. Diabetes insipidus: to treat or not to treat? Int J Radiat Oncol Biol Phys. (2021) 109:651–2. doi: 10.1016/j.ijrobp.2019.09.024 33516432

[35] Vakharia JD Thaweethai T Licht P Wexler DJ Delahanty LM. Psychological and behavioral predictors of weight loss in the reach ahead for lifestyle and health-diabetes lifestyle intervention cohort. J Acad Nutr Diet. (2023) 123:1033–43. doi: 10.1016/j.jand.2023.02.018 PMC11010584 36871848

[36] Akil H Burgess J Nevitt S Harding SP Alam U Burgess P. Early worsening of retinopathy in type 1 and type 2 diabetes after rapid improvement in glycaemic control: A systematic review. Diabetes Ther. (2022) 13:1–23. doi: 10.1007/s13300-021-01190-z PMC8776958 34928488

